# Specifically Targeted Transport of Plasma Membrane Transporters: From Potential Mechanisms for Regulating Cell Health or Disease to Applications

**DOI:** 10.3390/membranes11100736

**Published:** 2021-09-27

**Authors:** Yeqing He, Guandi He, Tengbing He

**Affiliations:** 1 College of Agricultural, Guizhou University, Guiyang 550025, China; gs.heyq20@gzu.edu.cn (Y.H.); tbhe@gzu.edu.cn (T.H.); 2 Institute of New Rural Development, Guizhou University, Guiyang 550025, China

**Keywords:** membrane transporters, target site recognition, targeted drug design, treatment of disease

## Abstract

Normal substrate transport and signal transmission are the premise to ensure the health of biological somatic cells. Therefore, a comprehensive understanding of the molecular mechanism of intercellular substrate transport is of great significance for clinical treatment. In order to better understand the membrane protein through its interaction with receptors, to help maintain a healthy cell and the molecular mechanisms of disease, in this paper, we seek to clarify, first of all, the recognition mechanism for different types of membrane protein receptors; pathogen invasion using the transport pathway involved in the membrane; and the latest specific target sites of various kinds of membrane transport carriers; to provide an explanation and summary of the system. Secondly, the downstream receptor proteins and specific substrates of different membrane transporters were classified systematically; the functional differences of different subclasses and their relationship with intracellular transport disorders were analyzed to further explore the potential relationship between cell transport disorders and diseases. Finally, the paper summarizes the use of membrane transporter-specific targets for drug design and development from the latest research results; it points out the transporter-related results in disease treatment; the application prospects and the direction for drug development and disease treatment providing a new train of thought; also for disease-specific targeted therapy, it provides a certain reference value.

## 1. Introduction

With the continuous development of basic medical research and clinical treatment technology, the etiology of diseases has been further and further studied, the relationship between cell transport and diseases has been gradually clarified, and specific targeted-therapy technology has been constantly updated [1]. The related theoretical research and drug development have been of wide concern to scholars at home and abroad. Most cell transportation is normal and effective, but if the body is invaded by some special pathogens, cell transportation will meet with obstacles, resulting in metabolic disorders, signal exchange disorder, the abnormal accumulation of substances, cell apoptosis and even carcinogenesis, which can then affect the normal metabolism of the body and even lead to death. This complex physiological process makes the treatment of and drug development for such diseases very difficult. Therefore, the premise for solving this important scientific question is to gain a better understanding of the molecular pathways that sustain the specific transport of membrane transporters in organisms.

Cell membranes, including membrane carrier proteins, membrane channel proteins and ATP drive pumps, are the main transporters [2]. Membrane transporters have wide, but specific tissue distributions. They can impact on multiple endogenous and xenobiotic processes [3,4]. Transport proteins constitute approximately 10% of most proteomes and play vital roles in the translocation of solutes across the membranes of all organisms [5]. The receptor proteins on the cell membrane are also important structures involved in substrate transport and signal communication. The obstacles of cell transport-related proteins directly lead to the lack or excess of certain substances in cells. This can cause a series of chain reactions, resulting in serious diseases in the body. Cell receptor proteins are the structural basis of signal communication and the transport of macromolecules [6]. Abnormal signal receptors lead to abnormal signal communication. The abnormal excitability and signal activation of cells result in cell dysfunction [7]. Many studies have reported the structure and transport medium of cell transportation-related proteins. Studies have also analyzed the structure of abnormal proteins providing directions for the development of related drugs. A large number of therapeutic drugs have been developed and utilized. The proteins related to cell transport can participate in the transport of metabolic substances, and can also be used for unconventional drug methods. The structural analysis of proteins related to cell transport also provides targets for the treatment of many diseases and the delivery vectors of unconventional drugs and intracellular drugs.

Cells have a complicated transport process, but there are a lot of specific recognition receptors to be found on the membrane transporters which have gradually clarified the transport mechanisms underlying disease and the health status of cells, and the precision of their means of control for the development of targeting drugs on cells, and it is expected to apply basic theoretical study to clinical treatment. The latest research results on the specific targeting of the transport mechanism of plasma membrane transporters lack a systematic summary. Based on this, we have reviewed the latest research progress, including targeted recognition sites for different types of membrane transporters, specific substrate recognition and drug development. We hope to provide some ideas for the development and utilization of unconventional drugs.

## 2. Membrane Transporters

### 2.1. Carrier Proteins

Membrane carrier proteins are multiple transmembrane proteins and exist in almost all types of biofilms. Each carrier protein can transport a specific solute molecule through a series of conformations that mediate the transport of solute molecules across membranes and can participate in active and passive transportation. The plasma membrane of the cell has carrier proteins for the input of nutrients (sugars, amino acids, nucleotides, etc.). The mitochondrial intima contains carrier proteins that can input pyruvate and ADP and output ATP. Carrier proteins are highly selective in substrate transport, and usually only one class of molecules is transported. The transport of carrier proteins is similar to the process by which enzymes bind to substrates and the process becomes saturated. Some carrier proteins are pH dependent [8]. The properties of common biofilm carrier proteins are shown in Table 1.

### 2.2. Channel Proteins

**Figure 1:**
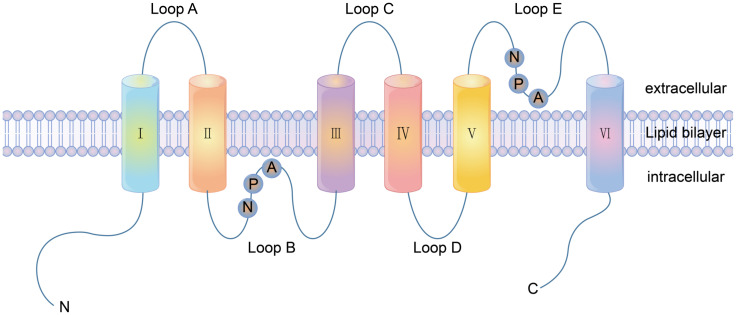
The structure of aquaporin AQP1 consisting of six transmembrane domains and five loops. A, C and E are located lateral to the plasma membrane, whilst B and D are located medial to the plasma membrane. Loop B and E are highly hydrophobic and have highly conserved ASN-pro-ALA (NPA repetitive tandem sequence), and are the passage regions of AQPs.

There are three types of channel proteins: ion channels, porins and aquaporins. Most of the channel proteins identified so far are ion channels [18]. Ion channels include electric pressure gated channels, ligand-gated channels and pressure activation channels [19]. The diameter and shape of the channel directly determine the selectivity of the ion passage, which does not involve the binding of the solute molecules. At present, more than 100 channel proteins have been discovered that are ubiquitous in the plasma membrane and the intima of various eukaryotic cells. The main classification of these is shown in Table 2. Porins are found in the outer membrane of Gram-negative bacteria and the outer membrane of mitochondria and chloroplasts. Porins have low selectivity and can pass through larger molecules. In 1988, Areg et al. accidentally discovered CHIP28, a 28KD hydrophobic membrane protein [20]. In 1997, CHIP28 was renamed Aquaporin 1 (AQP1) [21]. Aquaporins (AQPs) have been found in many plants, animals and microorganisms. AQPs are fast-growing cell membrane proteins that participate in transmembrane transport and the transport of important molecules by forming stomata as water channels. There are 13 subtypes and 3 subfunctional families of AQPs expressed in mammals, AQP0, AQP1 and AQP12. According to the different functions of each subtype in the body, the AQPs can be divided into three categories: pure AQPs, (AQP1, AQP2, AQP4, and AQP5), aquaporin channel proteins (AQP3, AQP9, AQP10) and super AQPs (AQP6, AQP8, AQP11 and AQP12) [22,23]. AQP1 was the first fully parsed aquaporin [24]. Loops A, C and E are the extracellular loops, B and D are the intracellular loops. The protein consists of two internal tandem repeats that roughly cover the amino and carboxyl ends of the protein. Each repeat consists of three transmembrane helixes and a highly conserved loop following a second transmembrane helixes (loop B and loop E). This loop includes a conserved characteristic motif, asparagine-proline-alanine (NPA). Loop B and E form short α helices that fold back into the cell membrane, with loop B entering the cell membrane from the cytoplasmic side and loop E entering the cell membrane from the outer side. Thus, a seventh transmembrane domain is formed, in which two NPA boxes are oriented at 180 degrees to each other to form water channels through the protein pores (Figure 1).

### 2.3. ATP-Driven Pumps

ATP-driven pumps are a family of proteins that rely on the energy released by ATP hydrolysis to transport substances across membranes, including the P-type ion pump, F-type proton pump, V-type proton pump and the ATP-binding cassette (ABC) transporter [30]. The characteristics of the four ATP-driven pumps are shown in Table 3. The p-type pump is located in the plasma membrane, and each P-type pump has two α subunits and two β subunits. The α subunit plays the transport function, and at least one β subunit participates in the phosphorylation process. This acts to regulate the pump activity, assist the transport of Na^+^, K^+^, H^+^, Ca^2+^, and jointly maintain the homeostasis of ions inside and outside the plasma membrane. Type F is mostly found in the inner membrane of the mitochondria, the chloroplast capsule membrane and the bacterial plasma membrane. It plays an important role in energy conversion and converts ADP into ATP by proton dynamic potential, and so is also called ATP synthase [31]. The V-type proton pump has a complex structure with multiple subunits and is involved in H^+^ transport. The transport process does not involve phosphorylation intermediates, and is involved in maintaining renal acid balance, regulating apoptosis and cell cycle, nerve signal transmission and other physiological functions in animals. The structure, function and mechanism of the ABC protein have always been the focus of research [32]. ABC transporters are composed of two highly hydrophobic transmembrane domains and two intracellular nucleotide binding domains. They bind together through hydrophobic interactions and are widely distributed in various organisms from bacteria to humans. The transport substrates include sugars, ions, amino acids, phospholipids, peptides, polysaccharides and even proteins [33].

### 2.4. Functions of Membrane Transporters

#### 2.4.1. Function of Carrier Protein

Carrier proteins are important proteins in the cell membrane as they form channels for the transport of nutrients such as sugars, amino acids, nucleotides and other small molecules to enter and exit the cell. Amino acid carriers in mammalian cells can be divided into neutral, acidic, alkaline transporters, and Na^+^ dependent and Na^+^ independent carriers, which are involved in amino acid transport in cells [39]. Amino acid transporters also participate in the regulation of intracellular signaling pathways, including the mechanistic target of rapamycin complex 1 (mTORC1) and general control nonderepressible 2 (GCN2), and participate in physiological homeostasis and nutritional health regulation [40]. Glucose uptake in mammalian cells is assisted by glucose transporter proteins. There are at least five such transporters that have different characteristics: the glucose transporter type 1 (GLUT1), GLUT2, GLUT3, GLUT4 and GLUT5. GLUT2 and GLUT4 are of great significance in diabetes. Sodium-glucose co-transporter (SGLT) is also the focus of research and plays a key role in intestinal glucose absorption [41].

#### 2.4.2. Function of Channel Proteins

AQPs exist in the form of a tetramer, and in which each monomer is involved in water transport. AQPs can participate in the absorption and secretion of water to maintain a balance of body water, electrolyte metabolism, cerebrospinal fluid secretion, urine dilution and intestinal water [42]. AQP is also involved in tumor growth, invasion, and metastasis, gastrointestinal disease, and kidney disease [43,44]. The functions of ion channels include protein kinase activation and gene expression regulation, the regulation of cell excitability and muscle activity, and maintaining cell volume. Ion channels play an important physiological role in the body that is directly related to homeostasis.

## 3. Membrane Receptor Proteins

### 3.1. Types of Membrane Receptor Proteins

#### 3.1.1. Ion Channel-Coupled Receptors

**Figure 2:**
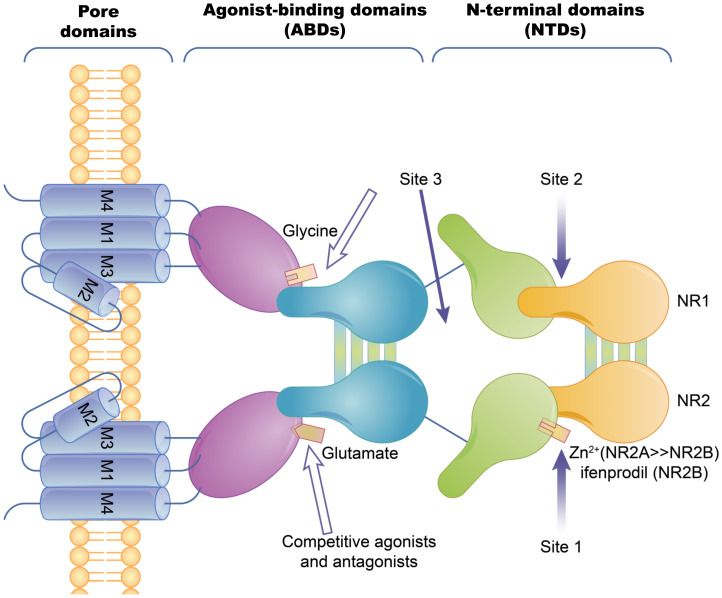
The basic structure of the NMDA ion-channel receptor. The NMDA receptor is composed of two NR1 subunits and two NR2 subunits in a dimer combination. Only one of the NR1/NR2 heterodimers is shown. The extracellular region of each subunit has two projections, the N-terminal and the agonist binding region, where the polymerization of subunits occurs at both the agonist binding region and the N-terminus. Agonist binding sites with glutamic acid are present in the NR2 subunit and glycine or serine binding sites are present in the NR1 subunit. Hollow arrows indicate the binding sites of competitive agonists or antagonists, and solid arrows indicate the regulatory sites for allosteric conditioning (e.g., zinc ions).

Ion channel-coupled receptors are a class of macromolecules that are ion channels and have ligand-binding sites, which can act as ion channels and signal transducers. Different from ion channels controlled by potential and chemically modified ion channels, the opening and closing of these ion channels are directly controlled by ligands, which are mainly neurotransmitters [45]. Ion-channel receptors can be cation channels, such as those for acetylcholine, glutamate, and serotonin, or anionic channels, such as those for glycine and gamma-aminobutyric acid [46]. Taking the N-methyl-D-Aspartic Acid receptor (NMDA) as an example, ion channels can be blocked by different channel blockers. The NMDA receptors can be blocked by endogenous magnesium ions, MK-801, and noncompetitive antagonists, such as memantine and ketamine [47]. The known subtype-selective NMDA receptor antagonists are zinc ions that bind to the N-terminal of the NR2 subunit (acting on the NMDA receptor containing the NR2A subunit) and Benz phenols that selectively block the NMDA receptor containing the NR2B subunit (Figure 2).

#### 3.1.2. G Protein-Coupled Receptors

**Figure 3:**
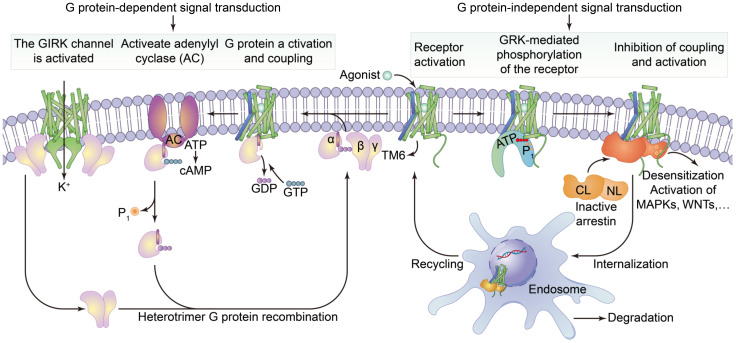
The structure of G protein-coupled receptors and downstream signal transduction pathways. It shows that the agonist binds to activate the receptor by inducing a conformational change in the transmembrane domain (TM6, blue). The activated receptor binds to a variety of intracellular signaling proteins, including G proteins (light purple), and GRKs (light orange), that are active (yellow) and inactive (dark orange). The coupling of heterotrimer G proteins to the receptor initiates nucleotide exchange, and then the G proteins dissociate into the Gα, Gβ and Gγ subunits. Both subunits regulate different downstream effector proteins. The GTP-bound Gα subunit regulates the activity of adenylate cyclase (AC, dark purple), whilst the Gβ and γ subunits interact with the g-protein-coupled internal rectifying potassium channel (GIRK, represented by cylindrical TM, green). The G protein-mediated signaling pathway is terminated by GTP hydrolysis and the recombination of Gα with Gβ and γ to form inactive heterotrimers. Activation of the receptor also leads to phosphorylation of GRKs and subsequent coupling of statin. Statin-coupled receptors lead to desensitization and statin-mediated activation of downstream effector proteins, such as mitogen-activated protein kinases (MAPKs) or SRC kinases. Statin activation also promotes receptor internalization into the endosome and subsequent degradation or circulation into the plasma membrane.

Cell surface transmembrane proteins are mainly composed of G-protein-coupled receptors (GPCRs), ion channels and transporters, which play an important role in neuronal signal processing and plasticity in the brain. The function of GPCRs in cell motility, growth differentiation and gene expression is closely related to tumors. The GPCRs are the largest family of cell-surface receptors and are ubiquitous on the surfaces of various eukaryotic cells. Conjugated GPCR proteins also mediate different signaling pathways [48]. As shown in Figure 3, GPCRs include a variety of neurotransmitters, peptide hormones and C-X-C chemokine receptor (CXCR), and receptors that accept exogenous physical and chemical factors in the senses of taste, vision and smell. CXCR4 plays a key role in tumor invasion and metastasis. CXCR can induce neutrophils, lymphocytes, monocytes and fibroblasts to aggregate and activate the inflammatory sites and participate in tissue injury repair. CXCR is important member of the GPCR family, it mainly causes downstream signal transmission. G proteins regulate ion channels through second messengers, and the activity of many ion channels is influenced by specific GPCR activation. Phosphatidylinositol signaling pathway is an extracellular signal molecule that binds to GPCRs on the cell surface, activates phospholipase C on the plasma membrane, hydrolyzes phosphatidylinositol bisphosphate (PIP_2_) on the plasma membrane into the inositol 1,4,5-trisphosphate (IP_3_) and diacylglycerol (DG), and converts extracellular signals into intracellular signals. The phosphatidylinositol-3-kinase/protein kinase B/mammalian target of rapamycin (PI3K/Akt/mTOR) signaling pathway is considered to be involved in the regulation of cellular physiological processes through the activation of downstream effector factors, and is directly related to cell growth, proliferation, cancer and longevity. This pathway is involved in the occurrence of human diseases and can regulate many biological functions in the body [49]. cGMP activates cGMP-dependent protein kinase PKG and downstream MAPK pathways, resulting in tumor cell dryness and metastasis [50]. Structurally, these receptors are monomer proteins with the amino terminus on the outer surface of the cell and the carboxyl terminus on the inner membrane. The complete peptide chain crosses the membrane seven times and so this type of receptor is also called a sevenfold transmembrane receptor. As the peptide chain repeatedly crosses the membrane, several loop structures are formed on the outer and inner sides of the membrane. These are responsible for binding to ligands (chemical and physical signals) and intracellular signal transmission, respectively. The cytoplasmic portion interacts with a GTP-binding protein (G protein), which is the first signaling molecule in the pathway [51]. G protein-coupled receptors regulate a variety of intracellular signaling cascades including G protein-dependent and G protein-independent pathways [52].

#### 3.1.3. Enzyme-Linked Receptors

**Figure 4:**
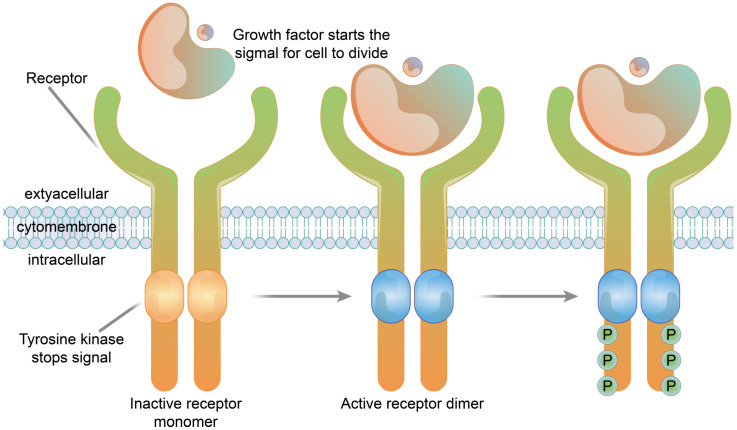
The structure of a receptor tyrosine kinase. In the absence of ligand binding, the receptor is combined with two monomers and is not active. The signaling molecule and the extracellular receptor combine to form dimers on the membrane. The tail end of the two receptors and the intracellular structure domain interact to activate the protein kinase function, which causes the phosphorylation of tail tyrosine residues. Phosphorylation causes the tail of the receptor’s intracellular domain to assemble into a signaling complex. The phosphorylated tyrosine site becomes the binding site for intracellular signaling proteins. Ten to twenty different intracellular signaling proteins can bind to the phosphorylated site of the receptor tail and are activated. Signal complexes amplify the information and activate a series of biochemical reactions in cells through several different signal transduction pathways, or combine different pieces of information to produce a comprehensive response.

The receptors of many growth factors and cytokines have structures with single transmembrane glycoproteins, enzyme activities or involve the intracellular segment of the receptor associated with the enzyme. In contrast to the seven-fold transmembrane receptors (G protein-coupled-type receptors), these receptors are referred to as single-fold transmembrane receptors, and are also known as enzyme-linked receptors. The transmembrane regions of these receptors cross the membrane once, in contrast to the structure of seven-fold transmembrane receptors which have repeated transmembrane segments. Receptor tyrosine kinases are typical examples of enzyme-linked receptors [53]. Immunosorbent assay (Cell-ELISA) analysis of native and recombinant glutamate receptors, including growth factors and cytokines, such as growth hormone receptor, interleukin receptor, and tumor necrosis factor receptor, etc., (Figure 4).

### 3.2. Function of Membrane Receptor Proteins

#### 3.2.1. Ion Channel Receptor Functions

The ultimate role of the ion-channel receptor signal transduction is to cause changes in the cell membrane potential. Ion-channel receptors affect the function of cells by transforming chemical signals into electrical signals [54]. The rapid conduction of electrical signals is transformed into chemical signal transmission, and then into electrical signals or cellular reactions to realize the rapid transformation of external signals [55]. Ion channel receptors are closely related to neural activity and muscle movement, and directly affect physiological activity in the body.

#### 3.2.2. Function of G Protein-Coupled Receptors

GPCRs are the largest family of cell signal transducers in the human body. GPCRs are involved in almost all physiological life activities and are responsible for regulating the responses of cells to light, odors, hormones, neurotransmitters and chemokines [56,57]. The extracellular regions of the GPCRs undergo conformational changes after binding to excitatory signaling molecules (e.g., odors, hormones, neurotransmitters, chemokines), leading to transmembrane helix movements, especially in the intracellular portion of the sixth transmembrane helix that turns outwards [58]. At this time, the intracellular region of the activated receptor can recruit and bind downstream effector proteins (such as G protein and B-Arrestin, etc.), that regulate physiological activities in vivo through the cyclic adenosine monophosphate (cAMP) signaling, phosphatidylinositol signaling and calcium ion signaling pathways [59].

#### 3.2.3. Enzyme-Linked Receptor Functions

Enzyme-linked receptor proteins are both receptors and enzymes. Once activated by ligands, the receptors have enzyme activities and amplify signals, and so they are also called catalytic receptors. This type of receptor transduction signal is usually related to cell growth, reproduction, differentiation and survival [60]. Receptor tyrosine kinases are typical enzyme-linked receptors. Extracellular ligands are soluble or membrane-bound peptides or hormones that include a variety of growth factors such as insulin, etc. The main function of enzyme-linked receptor proteins is to control cell growth and differentiation rather than regulating intermediate metabolism. Receptor tyrosine kinases are widely expressed in a variety of mammalian tissues and play important biological functions in the nervous, immune, hematopoietic and urinary systems [61].

## 4. Association between Membrane Proteins and Disease

### 4.1. Abnormal Ion Channels Induce Cancer

Ion-channel proteins are composed of protein complexes that allow suitably sized ions to pass along the concentration gradient. Studies have found that the K^+^ channel plays an important role in cell conduction, which is closely related to many diseases and plays an important role in the study of tumors and cancer [62]. The K^+^ channel protein promotes Ca^2+^ influx and G1 phase progression by influencing tumor cell membrane potential, or by changing cell volume, the concentration of intracellular substances related to DNA synthesis and cell cycle regulation can be affected, thus causing the rapid proliferation of tumor cells [63,64]. In addition, K^+^ channel protein expression is also associated with tumor metastasis, for example, overexpression of the G protein-gated inwardly rectifying potassium (GIRK) is associated with lymph node metastasis of breast cancer [65]. Inhibition of K^+^ channel activity by chemical reagents can inhibit the proliferation of cancer cells, indicating that the K^+^ channel protein can be used as a drug target.

The Na^+^ channel is a kind of transmembrane glycoprotein located in the plasma membrane of the cell, its main function is to maintain cell excitability and conduction. The selective Na^+^ permeability of voltage-gated Na^+^ channels is the basis of action potential generation in excitatory cells such as neurons. Bennett et al. found that prostate cancer cells with a high expression level of voltage-gate sodium channel and strong activity had better aggressiveness [66]. In breast cancer, cervical cancer and melanoma, it has been found that upregulation of the Na+ channel can promote tumor metastasis.

The Ca^2+^ channel is involved in intracellular regulation of almost all biological functions of the body, such as heart and muscle contraction, nerve information transmission, cell proliferation and apoptosis, cell division and differentiation, etc. [67]. Studies have found that the store-operated Ca^2+^ entry (SOCE), as an important pathway to regulate intracellular calcium homeostasis, is closely related to tumorigenesis and plays an important role in maintaining calcium homeostasis in angiogenesis and tumor immunogenicity changes [68].

Na^+^/K^+^-atPase (NKA) utilizes the hydrolysis of ATP to actively transport Na^+^ and K^+^ across the membrane, against the concentration gradient to transport Na^+^ to the extracellular and K^+^ to the intracellular, which can maintain cell membrane localization and make the cell present an excited state [69].

### 4.2. Substrate Transport Disorders, Induced Metabolic Disorders of the Type of Disease

Membrane transporters have different structures and physiological functions. Gene mutations usually produce defective proteins that cause abnormal transport channels. This normally results from the loss of normal function due to functional enhancement, functional loss and dominant and negative effects. Any kind of membrane transport-related protein disorder may cause abnormal physiological states in cells, resulting in cellular substrate transport disorders and cell body diseases. The cytoplasmic membrane has selective permeability, which plays an important role in cell substrate metabolism and osmotic pressure maintenance. Carrier proteins are transmembrane proteins with multiple cyclotron folds that bind specifically to the molecule being delivered to allow it to cross the plasma membrane. Their functional mutations or deletions can lead to a series of diseases related to material transport, as summarized below.

GLUT1 deficiency is a metabolic disease of the brain that is caused by mutations in the SLC2A1 gene. Up to now, more than 140 different SLC2A1 variants have been found, all of which can lead to decreased expression or the complete loss of GLUT1 function, impaired glucose transport in the brain, and insufficient energy supply [70,71]. Mutations of the GLUT1 protein lead to misfolding and polymerization resulting in impaired ACTIVITY of GLUT1 [72]. Cystinuria is a disease characterized by specific defects in the transport of cystine, lysine, arginine and ornithine in hereditary proximal renal tubular epithelial cells and the jejunal mucosa. Cystinuria results in the excessive excretion of these four amino acids in urine. Lysine, arginine, and ornithine are easily soluble in water, whilst cystine is less soluble in water, cystine has low solubility in urine and can crystallize to form stones [73]. AQP maintains renal urine concentration through the cell transport of water and low molecular solutes. Mutations or functional defects in AQP genes may lead to severe nephrogenic diabetes insipidus. A large number of studies have shown that AQPs are associated with acute kidney injury and various chronic kidney diseases [74,75].

The dysfunction of the ATP-binding cassette (ABC) transporter proteins can lead to several human diseases. More than 20 ABC transporters are known to be associated with human diseases, ABCA4 is linked to Stargardt disease. ABCA7 is linked to Alzheimer’s disease, two-thirds of ABCB lesions lead to immune deficiency and the lesions of ABCC7 can lead to cystic fibrosis [76,77]. P-glycoprotein (P-gp), Multidrug Resistance protein-1 (MRP1) and ABC superfamily G member 2 (ABCG2) transporters are expressed at abnormally high levels in the cell membranes of tumor cells [78]. P-gp plays an important role in the exogenous defense system, which can transport organic cations, carbohydrates, antibiotics and anticancer drugs as substrates [79]. Studies have shown that MRP1 is resistant to some cancer drugs, such as anthracyclines, vinblastine alkaloids, and campinetin, which is a major challenge in the clinical treatment of neuroblastoma [80]. Sodium-glucose co-transporter-2 inhibitors (SGLT-2i) mediated glycosuria decreases with a decrease in blood glucose concentration and renal glucose threshold, and the risk of hypoglycemia does not occur with medication alone. Therefore, due to its unique mechanism of action, SGLT-2i has become an important new target drug for the treatment of the type 2 diabetes mellitus (T2DM) and diabetic nephropathy (DN) [81]. ABCG2, known as breast cancer resistance protein (BCRP), can make cancer cells resistant to a variety of drugs, such as topotecan, mitoxantrone and daunorubicin [82].

### 4.3. Membrane Receptors and Pathogen Invasion

Pathogens invade cells by combining with specific receptors on the cell surface. Under normal circumstances, receptors on the cell surface can distinguish between pathogens and normal substances within the body. When encountering pathogens, cells stimulate autoimmunity and kill the pathogens. However, some pathogens can camouflage their surface structures, and evade cell recognition through the endocytosis pathway.

In the case of HIV, HIV requires CD4 receptors to enter cells. It also requires co-receptors such as cinnamoyl-COA reductase (CCR) or CXCR. Macrophages and dendritic cells have CD4 and CCR5 receptors, and CD4T lymphocytes have CD4 and CXCR4 receptors. The entry of HIV into human cells requires the participation of CD4, CCR5 or CXCR4 receptors, otherwise it cannot cause infection [83]. Studies have shown that people who are resistant to HIV have a deletion mutation in their CCR5 receptor gene, that results in a 32-base pair loss. In these cases, the normal CCR5 receptor protein cannot be encoded and so macrophages and dendritic cells lacking the CCR5 cannot be infected by the virus and individuals are resistant to AIDS [84]. Since the outbreak of the novel coronavirus, the mechanism of the invasion of human cells by COVID-19 has become clear. To invade human cells, the novel coronavirus must catch the angiotensin-converting enzyme 2 (ACE2) protein on the surface of human cell membrane with the help of the S protein on its surface. The virus binds to these proteins before entering human cells and causing infection [85].

## 5. Membrane Proteins and Their Applications in the Treatment of Diseases

### 5.1. Theoretical Basis of Disease Treatment

#### 5.1.1. Biotherapy Vector Recognition Sites

Biotherapy is a new treatment method, at present, using membrane-like micro/nano-structures, mainly composed of liposomes and nanospheres, as popular carriers for drug delivery. The accurate identification and internalization of the treatment carriers are the focus of current research, and are particularly important in improving targeted drug delivery. There are many recognition sites on the cell surface, and the ligand design based on the structural characteristics of the receptors is the main direction being taken to improve targeted drug delivery. As a natural drug delivery system, extracellular vesicles (EVs), have multiple ligand/receptor-modified lipid bilayer membranes. These can interact with targeted cells. Exosome integrin can interact with specific molecules on vascular cells mediating the exocytosis of inflammatory sites [86]. Liposomes have a similar structure to EVs and can be prepared artificially. Cell technologies and artificial expression vector construction have allowed surface receptor targeting and the synthesis of liposomes as therapeutic vectors [87].

#### 5.1.2. Extracellular Targets for Drug Therapy

The cell is a complex organism that responds to different microenvironments. As a stimulus signal, drugs can also produce different effects through different targets in different microenvironments and pathological conditions. Membrane transporters enable efficient cellular metabolism, aid in nutrient sensing, and have been associated with various diseases, such as obesity and cancer [88]. Drug transporters are a series of proteins that exist on the membrane surface of tissues and organs and act as drug transmembrane transport. Their main functions include the active transfer of drugs into organisms, the effective distribution of drugs into the target organs of organisms through the mediation of capillary endothelial cells and the membrane surface transporters of various organs, and finally the excretion of drugs and their metabolites through liver and kidney in vivo [89,90]. Transporters that mediate drug entry into cells can fully absorb substrates to the target for drug effect. They belong to the solute carrier (SLC) family and mainly include the L-type amino transporter (LAT), peptide transporters (PEPTs), sodium dependent secondary active transporters (SGLTs), GLUTs, monocarboxylate acid transporter (MCT) and the organic cation transporters (OCTs). The transporters that mediate drug delivery mainly include the P-gp, multidrug resistance associated proteins (MRPs), breast cancer resistance protein (BCPR) and the bile salt export pump (BESP), which belong to ABC family and can transport drugs and endogenous substances using the energy of hydrolyzed ATP [91,92]. In addition to the ABC transporters playing a crucial role in multidrug resistance in cancer, SLC transporters have recently been reported to be involved in multidrug resistance. The organic cation transporter 2 (OCT2) is the highest expressed ingested drug transporter in kidney and is directly related to multidrug resistance of renal cancer cells. Through bioinformatics studies, it was found that the decreased expression of OCT2 in renal cancer cells was closely related to the oxaliplatin resistance of renal cancer cells. The combination of DNA methyltransferase inhibitor decitabine and oxaliplatin reverses OCT2 underexpression in renal carcinoma cells and sensitizes the anticancer effect of oxaliplatin [93,94]. The treatment of abnormal membrane protein diseases can be based on its structural characteristics against treatment, competitive inhibition and other measures to reduce damage and relieve symptoms. Based on the surface structure covering of invading pathogens and substances, blocking the binding of pathogens to the surface of cell membranes is also an important way to inhibit pathogen infection [95,96]. The specific recognition of membrane proteins is a potential target of drug therapy, with more than 60% of drugs targeting membrane proteins. G protein-coupled receptors and ligand-gated ion channels are important drug targets that constitute 40% of the currently approved drugs. After the binding of extracellular ligands onto the cell membrane, intracellular signaling cascades are initiated. The traditional idea of drug development for these receptors is to promote or block their activation by directly targeting these receptor proteins [97]. Nowadays, drug transport-mediated drug interactions have attracted much attention. More and more drugs have been identified as specific substrates, inhibitors and inducers of transporters in vital organs of the body, as shown in Table 4.

### 5.2. Application of Membrane Proteins in Disease Treatment

In the treatment of malignant tumors, chemotherapy is an important method in addition to surgery. The use of small molecule antitumor drugs can effectively stop or slow the growth of malignant tumors. However, a large number of small molecule antitumor drugs do not have the ability to recognize tumor cells and will act on normal cells, leading to large adverse reactions [105]. Based on the needs of modern precision medicine, the development of tumor-targeted drugs has made great progress. Targeted therapy is an effective method to reduce the adverse reactions of small molecule antitumor drugs and improve the overall efficacy. After decades of development, the targeted delivery of drugs by nanodrug delivery systems (such as micelles, liposomes, etc.) has been realized. Precursor drugs are also an important strategy for tumor-targeted drug delivery [106].

#### 5.2.1. Recognition Sites for Nanodrug Delivery Carriers

Nanodrug delivery systems have been widely studied and reported in the literature. Polymer micelles with small particle sizes can easily pass through various barriers, and be easily surface modified to increase the accumulation of drugs at the target site [107,108]. Micellar drug loading can involve the use of lipid-soluble macromolecules as carriers, including insoluble drugs in the preparation of drug-loaded polymer micelles. Chemical condensation is used to anchor the targeted molecules on the surface of polymer micelles and to produce drug micelles for cell membrane targeting [109]. Zhang et al. [110] synthesized a polysialic acid/ursolic acid polymer (PSAU) by condensation reactions. To further test the drug-loading capacities, studies have investigated the preparation of a polysialic acid/ursolic acid amphiphilic copolymer micelle (PTX-loaded PSAU) loaded with paclitaxel using a nanoprecipitation method. In this study, PTX-loaded PSAU micelles showed excellent stability at physiological pH and the PSA was easily hydrolyzed under slightly acidic tumor conditions, so PSAU was able to release the loaded PTX at the tumor site. In vitro cytotoxicity studies showed that PSAU micelles were minimally cytotoxic to gastric adenocarcinoma cells, whilst PTX loaded micelles showed high cytotoxicity [111]. In this study, ursolic acid micelles acted not as independent antitumor agents, but as a novel drug delivery system to enable traditional chemotherapeutic agents to function better [112,113,114]. However, at present, the main problems affecting micelle applications are associated with small drug loading and difficult carriers that cannot be mass-produced.

**Figure 5:**
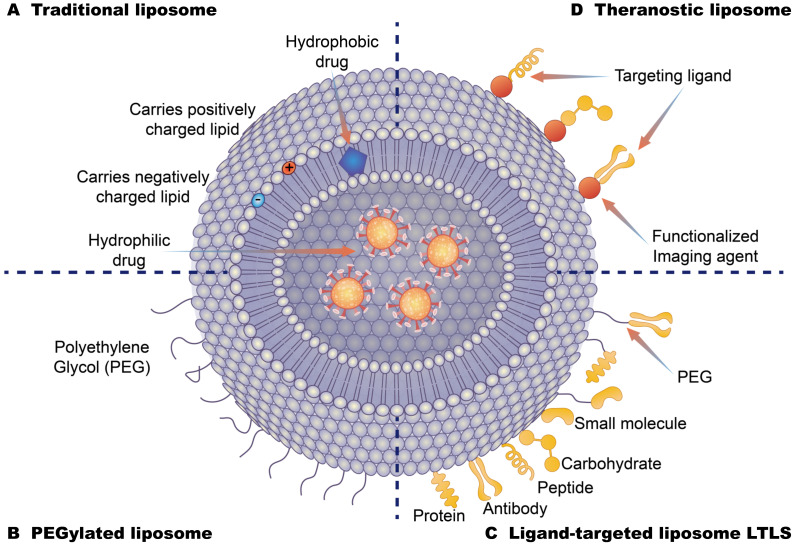
Different types of liposome drug-loading strategies (**A**): Traditional liposomes are based on the phospholipid bilayer. Hydrophobic drugs are loaded into the middle of the bilayer, and hydrophilic drugs are loaded on the internal surface of liposomes with positive and negative potential lipid molecules. (**B**): Polyethylene glycol liposomes are covered by polyethylene glycol on the surface of the liposome. These cover the hydrophobic layer of the liposome to extend the circulation time of the liposome in the body. (**C**): Ligand-targeting liposomes are bound to the surface of liposomes by specific targeting molecules to enhance the targeting of liposomes. (**D**): Therapeutic liposomes are composed of nanoparticles, target units, contrast agent components, and therapeutic agent components.

Drug-loading liposome technology has made great breakthroughs, many research studies have shown that liposome drug components can overcome the obstacles imposed by cells, in order to absorb and improve targeted distribution and other advantages, and these features ensure the drug composition of the liposome coating can be targeted for delivery to specific parts of the human body, thus improving effectiveness and minimizing side effects. At present, liposome drug delivery systems can be divided into several categories (Figure 5). Class A systems include traditional liposomes, which are composed of ionized phospholipid lipids, cholesterol and the aqueous phase core that is formed by the aqueous phase and the phospholipid layer which can contain water-soluble and fat-soluble components, respectively. Traditional liposomes can enhance drug delivery to diseased tissues by altering drug metabolism and biodistribution, thereby reducing the biotoxicity of drug components in vivo. However, these liposome delivery systems are easily and rapidly cleared in the blood flow with limited efficacy [115,116]. Class B systems are pegylated liposomes. The behavior on liposomes in vivo, especially in blood, can be modified by surface modification of polyethylene glycol (PEG) hydrophilic layer. PEG-surface modification can form a spatial barrier on the hydrophilic layer of the outer layer of the liposomes. This hydrophilic spatial barrier can weaken the effect of serum opsonin in vivo and avoid capture by the reticuloendothelial system, thus improving the spatial stability of liposomes and increasing the circulation times of drug-carrying liposomes in the blood. These changes can act to prolong the blood circulation times and also increase the accumulation of drugs at the lesion site and reduce the side effects of drugs [117,118]. PEG-modified long-cycle liposomes can improve the passive targeting of liposomes, and have been widely used in the development of liposomal pharmaceuticals [119]. Class C systems are ligand-targeting liposomes that graft recognition molecules (ligands) onto the surface or the end of PEG long chains. The specificity of ligands and interactions with receptor molecules on the surface of target cells can allow liposomes to release drugs in target areas. These liposomes use targeted ligands coupled to the liposome surface to selectively deliver drugs to the target site and are often referred to as “actively targeted” carriers. These drugs include antibody molecules, antibody fragments, and natural or synthetic ligands of small molecular weight moieties (such as folic acid, peptides, carbohydrates, glycoproteins). The selection of different ligands that are connected with liposomes can be targeted to different receptor-positive targets. The ligands of liposomes can be selected according to the needs of drug administration [120,121,122]. Class D systems are integrated therapeutic liposomes composed of nanoparticles, target units and contrast agents. These therapeutic agent components can be constructed on the basis of the previous three types of liposomes. For example, the integration of targeted delivery agents for diagnosis and treatment can be achieved by coupling precisely targeted antibodies to the surface of pegylated long-circulating liposomes and simultaneously loading drugs and labeled nuclides [123,124,125].

#### 5.2.2. Precursor Drug Recognition Sites

**Figure 6:**
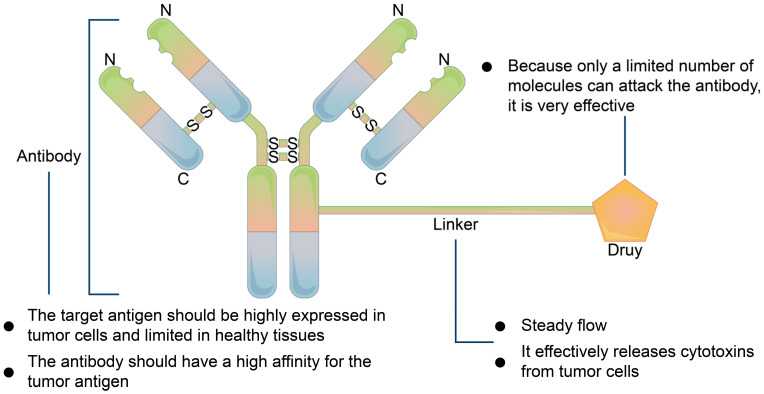
Basic mechanism of antibody-drug compound. The antibody must be highly expressed in tumor cells and limited in normal cells and have a high affinity for the tumor cell receptor. Linker, which must be stable in systemic circulation and can effectively release toxic factors from tumor cells when reaching tumor cells.

Precursor drugs have no biological activity or very low activity and become active substances after metabolism in vivo. The purpose of this process is to increase the bioavailability of drugs, strengthen targeting, and reduce the toxicity and side effects of drugs. The emergence of monoclonal antibody (mAb)-based immunotherapy has brought new ideas for targeted therapies. ADCs can selectively kill tumor cells by combining cytotoxin drugs with mAbs to deliver cytotoxin to tumor sites. The conjugation of an ADC cytotoxin, which is hydrophobic, and a mAb, which is hydrophilic, increases the possibility of drug polymerization. The biological function of mAbs is mainly determined by their structure. Antibodies are composed of antigen-binding domain (Fab) and crystallization domain (Fc). Fab specifically recognizes tumor-associated antigens and regulates downstream signaling pathways. Fc binds to the newborn IgG transporter (FcRn) and is not easily cleared in vivo, thus prolonging its half-life in the body [126]. Antibody-drug conjugates (ADC) are precursor drugs that are being widely studied at present, with monoclonal antibodies, as ligands of drug targeting sites, to guide drug targeting (Figure 6). A linker forms between an antibody and a drug. Small molecule drugs exert drug effects and some antibodies can work alone or in conjunction with other drugs. mAbs can reduce nonspecific toxicity by specifically binding antigens onto tumor cells, and by acting on specific signaling pathways to achieve therapeutic effect, or directly producing immune responses from tumor cells [127]. Currently, about 30 mAbs have been approved by the U.S. FDA for cancer treatments. To improve efficacy, mAbs have been covalently linked with various antitumor effector molecules (such as cytotoxic drugs, radionuclides and immunotoxins, etc.) to create targeted therapies and immunotherapies based on mAbs that have been widely studied. Gemtuzumab ozogamicin (Mylotarg) developed by Wyeth [128] was first used in clinical application, but it was later found that it could not improve patient survival and had significant side effects. Following Mylotarg, brentuximab vedotin (Adcetris) was approved in 2011 for the treatment of Hodgkin’s lymphoma and systemic mesenchymal large cell lymphoma [129]. T-dml (Kadcyla) was approved by the US FDA in 2013 for the treatment of HER2-positive metastatic breast cancer [130]. So far, seven drugs have been approved by the FDA, and more than 100 drugs have been used in clinical studies [131]. Despite major breakthroughs in drug development, the following problems still exist; the drug loading rate of antibodies is limited and it is difficult to determine the dosage and pharmacokinetics in vivo. The fracture connector breaks in advance in blood, so there is serious hepatotoxicity and other adverse reactions. Due to the limited penetration ability of tissues, it is difficult to treat solid tumors, and the miniaturization of antibodies should also be considered.

The design principles of polypeptide drug coupling are similar to that of monoclonal antibody coupling, which is mainly used for drug delivery and tumor targeting. The difference is that the antibody component in ADC is replaced by polypeptide molecules that can serve as targeted ligands [132]. Polypeptide conjugates have many advantages over antibody conjugates: as they are generally easier to prepare than pharmaceutical agents, are less costly, and are mostly nonimmunogenic [133].

Recently, aptamer drug coupling has been a focus of research. The systematic evolution of ligands by exponential enrichment (SELEX) techniques have identified single-stranded oligonucleotide libraries. Nucleic acid ligands that can bind to targets with high specificity and affinity also have many applications in the development of targeted therapies. Aptamers can form specific three-dimensional configurations (such as hairpins, convex loop and four-angle loops, etc.) through curling and folding. They can bind to targets through van der Waals forces, hydrogen bonds, electrostatic action and base loading forces, etc. The process is similar to the binding of antibodies and antigens and so aptamers are also called “chemical antibodies” [134]. Although similar in function to antibodies, aptamers have unique advantages as their binding affinity is comparable to that of most antibodies. In addition, compared with antibodies, aptamers are small, have low production costs, are easily chemically modifiable, have low immunogenicity, little difference between batches, high chemical stability, rapid tissue penetration, and are nontoxic [135,136].

## 6. Conclusions and Prospects

At present, some progress has been made in the analysis of the structure and function of molecules in cell transport. Abnormal cell transport in disease has become the focus of basic medicine research, and the development of drugs targeting the transport of diseases and other drugs based on transport structure have also made certain progress. However, cell transport is a complex and systematic process involving numerous molecular and biochemical reactions, and the current research is still insufficient. How to comprehensively understand the transport in cell health and disease is of great significance for basic science and clinical treatment.

Cell transport-related proteins play an important role in the cellular and systemic levels by the regulation of energy metabolism, protein synthesis, gene expression, REDOX balance, and signal transduction pathways. Cell transportation-related proteins are associated with a wide range of pathologies, including neurodegenerative diseases, inborn metabolic errors and chronic kidney disease, as well as hormone secretion and release, and their changes in function may be related to the pathogenesis of various diseases. Interactions between membrane transport proteins and with other proteins involve the functional regulation of substrate transport in terms of transport, localization, activity and degradation levels. This is a set of fine-grained regulatory mechanisms, in-depth study of which will help to fully understand the cellular transport mechanism of regulating organisms at the cellular level, and is expected to develop targeted therapeutics for cell-transport diseases. It is hoped that scientists can further clarify the structural characteristics of different types of transporters and the domain functions of unknown residues. These findings will provide valuable insights into fine-tuned cellular transport regulatory networks, transporter interaction models, and molecular probes for diagnosing diseases.

Membrane proteins play a role in cell transport and signal transduction, but can also be used as the targets for drugs. At present, the structural analysis of various membrane proteins in cells is not complete. How to find the protein differences between healthy cells and diseased cells, and how to design targeted drug therapies need further research to broaden the application range of targeted drugs. The variation of the cell membrane’s protein structure is the cause of many diseases. Targeted treatments can be achieved using gene editing, protein recombinant expression and other technologies. Similarly, rare diseases caused by abnormal cell membrane proteins are also topics that need to be focused on at present. New discoveries may be made by analyzing the etiology of various diseases.

## Figures and Tables

**Table 1:** Summary of the properties of common carrier proteins in animal biofilms.

Carrier Protein	Typical Positioning	Energy	Function	References
Na^+^-Glucose Pump	The apical plasma membrane of intestinal and renal cells	Na^+^	Glucose active transport	[9]
Na^+^-K^+^ Pump	The plasma membrane of most animal cells	ATP	Na^+^ active pumping and K^+^ active pumping	[10,11]
Na ^+^-H ^+^ Pump	The plasma membrane of animal cells	ATP	H^+^ active pumping	[12,13]
Na^+^ dependent neutral amino acid transporter	Absorbent epithelial cells	ATP	Amino acid pumping and downstream signal regulation of amino acid receptors	[14,15]
Na^+^ depends on a centralized carrier	Absorbent epithelial cells	ATP	Active transport of nucleosides	[16]
Glucose Carrier	The plasma membrane of most animal cells	—	Passive transport of glucose	[17]

**Table 2:** Classification of membrane channel proteins.

Species	Distribution	Somatotype	Function	References
Aquaporins	Brain; membranes; kidneys; testis; liver; nasopharynx; lungs; intestines; eyes; etc.	AQP0; AQP1; AQP2; AQP3; AQP4; AQP5; AQP6; AQP7; AQP8; AQP9; AQP10; AQP11; AQP12	Formation of various body fluids, reabsorption of water by tissues	[25,26]
Channel Protein	Chondriosome	MPTP	Apoptosis and necrosis	[27]
Ion Channel Protein	Various organizations	HCN; Slack; KcsA; TRPV; TRPM family; PKD1/2; PIEZO1/2; ENaC; TPCs; VDAC; SLC family; ASICs	Signal transduction, excitement transfer, substance synthesis, energy metabolism, osmotic pressure balance, nutrition induction, substance transport	[28,29]

**Table 3:** ATP-driven pump classification.

Kind	Constitute	Distribution	Function	References
P type ion Pump	2α subunits (transport), 2β subunits (regulatory)	Plasma membrane; endoplasmic reticulum	Na^+^, K^+^, H^+^ and Ca^2+^are transported across membranes	[34]
Type F ion Pump	Multiple subunits, transmembrane domain F0 and cytoplasmic domain F1	Mitochondrial inner membrane	ATP synthesis	[35]
V type ion Pump	Multiple subunits, transmembrane domain V0 and cytoplasmic domain V1	Intracellular bodies; lysosomal membranes; osteoclasts	H^+^ transport	[36]
ABC Transporter Superfamily	Two transmembrane domains, two intracellular ATP-binding domains	All kinds of organisms	Amino acids, sugars, lipids, peptides, protein transport, macromolecule transport	[37,38]

**Table 4:** Substrates, inhibitors and distributions of several common transporters.

Transporter	Gene Name	Substrates	Specific Inhibitors	Mainly Cells/Tissues/Organs	References
P-gp/MDR1	ABCB1	Operamide, quinidine, digoxin, fexofenadine, vinblastine, talinolol	Cyclosporine, quinidine, etc.	Blood-brain barrier, small intestinal epithelial cells, liver, tumor cells, kidneys, etc.	[98]
BCRP/MXR	ABCG2	Mitoxantrone, statins, anticancer drugs, etc.	Sulfasalazine, cyclosporin A, sulfasalazine, cyclosporin A, etc.	Hepatocytes, small intestinal epithelial cells, placenta, etc.	[99]
PEPT1	SLC15A1	Cefalexin, valacyclovir, ampicillin, amoxicillin	Cefalexin, valacyclovir, ampicillin, amoxicillin	Intestines, kidney	[100]
MRP2	ABCC2	Indinavir, cisplatin	Cyclosporin A	Intestines, liver, kidney, brain	[101]
OCT1	SLC22A1	Metformin, acyclovir, etc.	Midazolam, disopyramide, quinidine, etc.	Liver, intestines	[102]
MATE1 MATE2-K	SLC47A1/2	Cimetidine, zidovudine, metformin, etc.	Cimetidine, pyrimethamine, trimethoprim	Liver, kidney	[103,104]

## Data Availability

Not applicable.
